# A Simple Method of Preventing the Clouding of Mirrors

**Published:** 1898-02

**Authors:** Gilbert E. Seaman

**Affiliations:** Milwaukee, Wis.


### A Simple Method of Preventing the Clouding of Mirrors.

*To the Editor:*—The *Medical Press and Circular* recently called attention to an ingenious, effective, and at the same time very simple device for preventing the clouding of mirrors used in the work of laryngologists and dentists.

The method was introduced by George Wallis, L. D. S., dental surgeon to the London Throat and Ear Hospital, at a recent meeting of the staff.

It consists merely of smearing a thin layer of ordinary soap, soft but not moist, over the surface of the mirror, and afterward polishing the latter with a dry cloth. The effect of this procedure is that however much the mirror may be breathed upon, its reflecting surface remains perfectly clear and bright. When one takes into consideration the extreme simplicity of the procedure and the bothersome difficulties sometimes encountered in preventing the clouding of mirrors used in laryngology and dentistry, the value of this ready method can not be overestimated. It has proved very satisfactory to me, and as I have not yet seen it mentioned in the American journals, I take this opportunity of calling the attention of the profession to it.

Yours very truly,

GILBERT E. SEAMAN, M. D.

MILWAUKEE, WIS.

